# Drug-induced liver injury associated with Complementary and Alternative Medicine: a review of adverse event reports in an Asian community from 2009 to 2014

**DOI:** 10.1186/s12906-016-1168-z

**Published:** 2016-07-07

**Authors:** Desmond Chun Hwee Teo, Patricia Suet Leng Ng, Siew Har Tan, Adena Theen Lim, Dorothy Su Lin Toh, Sui Yung Chan, Han Hui Cheong

**Affiliations:** Department of Pharmacy, Faculty of Science, National University of Singapore, Block S4A Level 3, 18 Science Drive 4, Singapore, S117543 Republic of Singapore; Vigilance and Compliance Branch, Health Sciences Authority Singapore, 11 Biopolis Way, #11-03, Helios, Singapore, 138667 Singapore; Department of Pharmacy, KK Women’s and Children’s Hospital, 100 Bukit Timah Road, Singapore, 229899 Singapore

**Keywords:** Hepatotoxicity, Complementary and Alternative Medicine, Adverse event, Traditional Chinese Medicine

## Abstract

**Background:**

The use of Complementary and Alternative Medicine (CAM) has been increasing over the years. A recent review of adverse event reports (AERs) associated with CAM in Singapore found a notable number of AERs submitted. The objectives of this study are to analyse hepatotoxicity cases associated with CAM in Singapore based on spontaneous adverse event reporting to the Health Sciences Authority (HSA), and to highlight safety signals for specific herbal ingredients.

**Methods:**

AERs associated with CAM and hepatotoxicity submitted to the Vigilance and Compliance Branch (VCB) of the HSA from 2009 to 2014 were compiled. The following information was extracted and analysed: Demographic information; time to onset; hospitalisation status; outcome; type of hepatotoxicity; ingredients of CAM, and the total daily doses (TDD); concurrent western medicines and health supplements; and reporter details.

**Results:**

Fifty-seven reports were eligible for analysis. Thirty-five (61.4 %) cases involved Traditional Chinese Medicine (TCM). The Roussel Uclaf Causality Assessment Method was applied in 29 (82.9 %) of these cases, and the median score was 4 (range: 1–8). Chai Hu (*Radix bupleuri*) was suspected in 11 (31.4 %) cases. TDDs of most ingredients were within recommended doses of the Chinese Pharmacopoeia.

**Conclusions:**

Drug-induced liver injury is still poorly understood and more objective assessments are warranted. Reporting of adverse events should be strongly advocated to facilitate future analyses and the understanding of risk-benefit profiles of CAM.

## Background

CAM is a group of diverse medical and health care systems, practices, and products that are not presently considered to be part of conventional medicine [1]. Globally, CAM is used in up to 50 and 80 % of the population in developed and developing countries respectively [2, 3]. Its popularity has increased rapidly since the 1990s [4–6]. This expanding usage of CAM has been attributed to a number of factors [4, 6, 7], such as the increasing evidence of its efficacy in palliative care [8–11], the belief that it is a more holistic and well-rounded form of therapy [12, 13]; as well as a lower socioeconomic burden when compared to using western pharmaceuticals [14]. However, a high utilisation of CAM has also been associated with delays in seeking conventional or western, medical treatment [15].

In Singapore, CAM is broadly termed as Complementary Health Products (CHP), which consists three main categories: Chinese Proprietary Medicines (CPM), health supplements and other traditional medicines [16]. For the purpose of this study, the term CAM refers to CHPs. CAM use in Singapore was found to be high, in particular CPM and TCM, even amongst non-Chinese ethnic groups [17]. As of 31 Dec 2014, there were 10,344 CPM products listed in the database of the HSA, Singapore.

Under the Medicines Act of Singapore, CPMs refer to any medicinal products that have been manufactured into finished dosage forms (e.g. tablets, capsules) and used according to the TCM system of therapeutics [18]. These products can be from herbal, animal or mineral sources. Dealers who import, manufacture or market CPMs are required to list their products with the HSA, and ensure compliance with the regulatory requirements [19].

CAM, like western medicines, can also present with adverse effects, although they may generally be mistaken as safer than their western counterparts [7, 17, 20]. A study on AERs submitted to the HSA from 1998 to 2009 reviewed 627 cases associated with CAM and 80.2 % of these were found to be serious [21]. In previous studies of drug-induced liver injury (DILI) trends in Singapore conducted by Wai et al., CAM accounted for 22 out of 31 (71.0 %) and 15 out of 29 (52.0 %) cases of hepatotoxicity [22, 23]. This accentuates the possible severity of DILI related to CAM.

Diagnosis and clinical evaluation of DILI is particularly challenging, and has been a popular topic of debate amongst clinicians, academia, regulators and pharmaceutical companies [24]. Clinicians often rely on diagnostic markers such as aminotransferase levels and physical examinations to make an initial evaluation. Viral markers, history of alcoholism, signs of biliary obstruction, complications of underlying diseases such as sepsis, or chronic viral hepatitis are also typically evaluated in order to rule out other possible causes of liver injury. In spite of these, it is usually difficult to evaluate DILI appropriately [25]. Causality assessment becomes more complicated when DILI cases are also confounded by pre-existing medical conditions, concomitant western medicines, underlying liver pathology and other factors. Objective scales are thus utilised where possible to complement case evaluations.

The Roussel Uclaf Causality Assessment Method (RUCAM) was created based on the international DILI consensus criteria [26]. It applies numerical weightages to essential features of DILI in seven different domains [27, 28]. An overall score is generated and interpreted as a causality probability. The RUCAM is structured, quantitative, liver-specific, and validated for use in hepatotoxicity [29]. It has been shown to be more accurate and reproducible when compared to other causality assessment tools like the Naranjo scale [30, 31] and the Maria and Victorino scale [32].

This study aimed to analyse hepatotoxicity cases associated with the use of CAM, through a critical review of AERs in the Singapore’s National Pharmacovigilance Adverse Drug Reaction (ADR) database managed by the HSA. In particular, the ingredients of CPM and TCM products were analysed to identify any potential safety signals that could be associated with them. By studying these DILI cases, this study could better facilitate the surveillance of CAM products and promote their safe and effective use.

## Methods

This study was reviewed and approved by the National University of Singapore Institutional Review Board (NUS-IRB) (Approval number: NUS 2383; NUS-IRB reference code: B-14-229). A confidentiality agreement was also signed with the HSA for access to confidential information in the Pharmacovigilance ADR database and to safeguard the information received in the course of this project.

### Sources of data

AERs were submitted to the VCB of the HSA through the “Suspected Adverse Drug Reactions” form. The form contains multiple fields for entry by the health care professional or company reporting the adverse event, such as the patient’s demographic information, reporter’s details, details of the AE or ADR, management of the adverse event, as well as other relevant information. These reports were submitted by e-mail, facsimile, postal mail, or electronically through the Critical Medical Information Store (CMIS) component of the Electronic Medical Record Exchange (EMRX).

Submitted forms were reviewed by regulatory specialists of the VCB. These reports were examined for causality of the suspected drug or herb. Any information that was lacking or unclear were clarified with the reporters, and documented accordingly. All related materials and documents to each report were stored within the Pharmaceutical Regulatory Information System (PRISM) database. The information was also used in aggregate analysis. As a member of the World Health Organisation (WHO) International Drug Monitoring Programme, these reports are submitted to the Uppsala Monitoring Centre (UMC) in Sweden for collation into WHO’s VigiLyze [33].

### Data mining and e-collation

Adverse event(s) in each report were categorised using the WHO Adverse Reaction Terminology (WHO-ART). Filters were used to obtain the desired reports for the review. The System Organ Class chosen was “Liver and Biliary System Disorders”. “Complementary Medicines”, “CPM”, and “Health Supplements” were selected as the type of product involved, and the inclusion period was from 1st January 2009 to 31st December 2014 based on the date of receipt of the AER by the VCB. The WHO-UMC organisation system for standardised case causality assessment was also used. Only reports with the causality terms “certain”, “probable” or “possible”, as assessed by the VCB, were included for analysis. Reports that were associated with adulterated CAM products, assessed by the VCB as “unlikely” or “unconfirmed”, or had missing critical information such as ingredient lists or names of products, were excluded from the study. The statuses of all reports are updated as of 31st Jan 2015.

The following information were extracted from the reports and compiled into a Microsoft^®^ Excel spreadsheet: (1) Demographic information (i.e. age, gender, and ethnicity); (2) Date of onset and time to onset for the adverse event; (3) Hospitalisation status of the patient; (4) Outcome of the adverse event; (5) Type of hepatotoxicity involved; (6) Ingredients of all CAM products used, and the TDD of each ingredient; (7) Details of any concurrent western medicines; and (8) Profession of the reporter.

Hospitalisation status of the patients were categorised as “hospitalised”, “not hospitalised” and “already hospitalised”. The term “already hospitalised” describes patients who have been admitted for other co-morbidities prior to the adverse event. The outcomes of the events were classified into “recovered”, “not yet recovered”, “uncertain outcome” and “death”. The type of hepatotoxicity in the cases were classified according to the phenotypes of DILI, as described in the LiverTox database by the National Institute of Diabetes and Digestive and Kidney Diseases (NIDDK) [34]. A brief description of the different phenotypes is shown in Table 1. Western medicines that were consumed by the patient at the same time or within 3 months before the date of receipt of AER were considered as concomitant medications. To ascertain the likelihood of DILI in the cases for review, only CAM products that were used within the 3-month period prior to the date of receipt of AER were considered for analysis. The variables collected from the AERs were analysed using descriptive statistics.

**Table 1:** Phenotypes of Hepatotoxicity

Phenotypes of DILI	Brief description ^a^
Acute hepatic necrosis	Clinical course resembles an acute, toxic injury to the liver with sudden and precipitous onset, marked elevations in serum aminotransferase (ALT, AST) levels, and early signs of hepatic (or other organ) dysfunction or failure despite minimal or no jaundice. Rapid recovery after withdrawal of the agent is also typical. It is typically caused by a direct hepatotoxin and is usually dose dependent and “expected”, rather than idiosyncratic.
Acute hepatitis	Course of illness resembles acute viral hepatitis with insidious onset, a hepatocellular pattern of injury and jaundice. Illness typically lasts 2 to 4 weeks and ultimately resolves, but severe instances can result in acute liver failure and death.
Cholestatic hepatitis	Course of illness is marked by cholestasis, even early at the time of onset. The liver enzyme pattern is cholestatic with prominence of ALP and bilirubin elevations. The illness can be prolonged.
Mixed hepatocellular-cholestatic hepatitis	Course of DILI is considered “mixed” if features of both hepatocellular and cholestatic injury are present. The liver enzyme pattern is characterized by moderate to marked elevations in ALT, AST and ALP, such that the R ratio ^b^ is between 2 and 5.
Enzyme elevations without jaundice	The most commonly observed form of DILI is the elevation of ALT, AST or ALP (or all) without jaundice and with minimal or no symptoms.
Bland cholestasis	The course of illness is marked by prominent and typically prolonged jaundice and cholestasis with minimal serum enzyme elevations or evidence of hepatocellular necrosis.
Hepatic steatosis and lactic acidosis	The hallmark of this syndrome is hepatic microvesicular steatosis accompanied by lactic acidosis, with clinical and laboratory features of hepatic failure, such as encephalopathy.
Nonalcoholic fatty liver	Nonalcoholic fatty liver disease and steatohepatitis are well documented but rare forms of DILI. In addition, fatty liver disease is more often chronic than acute even when it is drug induced.
Chronic hepatitis	The course of illness resembles chronic viral hepatitis with serum aminotransferase elevations without jaundice and with mild symptoms if any. ALT and AST levels may fluctuate over time and intermittently fall into the normal range.
Sinusoidal obstruction syndrome	Sinusoidal obstruction syndrome, or veno-occlusive disease, is a distinctive and potentially fatal form of hepatic injury that occurs predominantly, if not only, after drug or toxin exposure.
Nodular regenerative hyperplasia	This condition typically presents with the insidious or unexpected onset of signs or symptoms of portal hypertension in a patient with little evidence of chronic liver disease.
Hepatic adenoma and hepatocellular carcinoma	Tumours of the liver include benign tumours such as hepatic adenomas, and malignant cancers such as hepatocellular carcinoma and cholangiocarcinoma.

*ALT* alanine aminotransferase, *AST* aspartate aminotransferase, *ALP* alkaline phosphatase, *DILI* drug-induced liver injury, *ULN* upper limit of normal

^a^ Descriptions of the various phenotypes of hepatotoxicity were adapted from the NIDDK LiverTox Database [34]

^b^ R ratio: A mathematical calculation to define whether the hepatic injury presented is “hepatocellular”, “mixed” or “cholestatic” in nature. Presented as $\frac{\frac{ALT}{ULN}}{\frac{ALP}{ULN}}$

Amongst the CAM ingredients compiled, those suspected to be associated with hepatotoxicity, either through expert opinion from the VCB or from previous documented reports in established databases such as LiverTox and the China Food and Drug Administration (CFDA), were further examined in detail. References to tertiary literature such as the Chinese Pharmacopoeia and textbooks on complementary medicines were also made. The RUCAM [27, 28] was employed to review the likelihood of these ingredients causing hepatotoxicity in the patients. The updated version of RUCAM was used to calculate the scores of the case reports [35, 36]. For cases with concomitant western medicines reported, the RUCAM was also used to evaluate the causality of those medicines with documented evidence of hepatotoxicity in the literature.

## Results

**Fig. 1:**
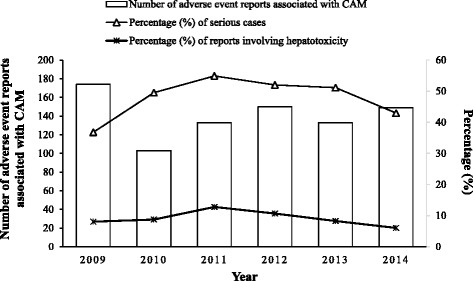
Number of AER associated with CAM received by HSA between 2009 and 2014. A total of 842 AERs associated with CAM were received by the HSA between the period of 2009 to 2014, of which 76 (9.0 %) involved hepatotoxicity

A total of 842 AERs associated with CAM were received by the HSA between the period of 2009 to 2014, of which 76 (9.0 %) involved hepatotoxicity. Based on the inclusion and exclusion criteria, 57 (75.0 %) of the 76 AERs involving liver injury were analysed. Figure 1 shows the year-on-year trend of AERs associated with CAM. On average, about 140 reported cases were linked to CAM every year, and about 9 % of these AERs involved hepatotoxicity.

There were 28 male (49.1 %) and 29 female (50.9 %) patients in the 57 liver injury case reports that were assessed. Majority (73.7 %) of them were above 40 years of age. Forty-seven (82.5 %) patients were of Chinese ethnicity. More than half (70.2 %) of the patients were hospitalised due to the adverse events. Thirty-three (57.9 %) patients had not recovered based on the latest report. Five (8.8 %) cases resulted in death. Demographics and other pertinent information extracted from the AERs are shown in Table 2.

**Table 2:** Demographic and characteristics of AERs received between 1^st^ Jan 2009 and 31^st^ Dec 2014

Demographics & information of patients	No. of cases, n (%)	Characteristics of adverse event reports	No. of cases, n (%)
1. Gender		5. Profession of Reporter of Adverse Event	
Female	29 (50.9)	Doctor	51 (89.5)
Male	28 (49.1)	Pharmacist	4 (7.0)
2. Age in years		Nurse	0 (0.0)
< 1	2 (3.5)	Drug Company	2 (3.5)
1–20	2 (3.5)	Others	0 (0.0)
21–40	11 (19.3)	6. Outcome of Adverse Event	
41–60	26 (45.6)	Recovered	12 (21.1)
> 60	16 (28.1)	Not recovered	33 (57.9)
3. Ethnicity		Death	5 (8.8)
Chinese	47 (82.5)	Uncertain Outcome	7 (12.3)
Malay	7 (12.3)	7. Type of CAM ^a^ implicated	
Indian	1 (1.8)	Traditional Chinese Medicine	35 (61.4)
Others	1 (1.8)	Health supplements	16 (28.1)
Information not available	1 (1.8)	Other Traditional Medicines	6 (10.5)
4. Hospitalisation Status			
Hospitalised	40 (70.2)		
Not hospitalised	12 (21.1)		
Already hospitalised	3 (5.3)		
Information not available	2 (3.5)		

Total number of reports included in data analysis, *N* = 57

Assumption: ^a^ 3 categories of CAM: (1) Traditional Chinese Medicine (includes both CPM and traditional Chinese remedies); (2) Health supplements; (3) Other traditional medicines (includes Malay Jamu and Indian Ayurveda). Cosmetic products were excluded

Most of the cases (61.4 %) involved TCM, followed by health supplements (28.1 %), and other traditional medicines (10.5 %) which included Malay Jamu and Indian Ayurveda products. For simplicity, the use of the term “TCM” here refers to both CPM and traditional Chinese remedies that are not finished products. Amongst the cases involving TCM products (*n* = 35), a total of 312 different Chinese herbal ingredients were compiled and analysed. The most common herbs found were Fu Ling (*Sclerotium poriae cocos*) (18 cases), Huang Qin (*Radix scutellaria baicalensis*) (15 cases), Gan Cao (*Radix glycyrrhizae*) (15 cases), Ze Xie (*Alisma orientalis*) (14 cases) and Chuan Xiong (*Rhizoma ligustici*) (14 cases). These herbs are described in Table 3.

**Table 3:** Most commonly found herbal ingredients with limited or lack of documented reports of possible hepatotoxicity

Name of herbal ingredient	No. of cases, n (%)	Types of hepatotoxicity implicated (n)	Mean TDD (g) (SD)	Mechanisms of action (based on TCM system of therapeutics) ^a^	Recommended daily doses (g) ^a^
Fu Ling *(Sclerotium poriaecocos)*	18 (31.6)	Acute hepatic necrosis (3), Acute hepatitis (11), Cholestatic hepatitis (1), Mixed hepatocellular-cholestatic hepatitis (1), Enzyme elevations without jaundice (2)	8.3 (13.4)	Promotes urination in order to drain dampness, strengthens the spleen and calms the heart.	10–15
Huang Qin (*Radix scutellaria baicalensis*)	15 (26.3)	Acute hepatic necrosis (3), Acute hepatitis (10), Mixed hepatocellular-cholestatic hepatitis (1), Enzyme elevations without jaundice (1)	6.3 (12.1)	Clears heat and dries dampness, purges fire to remove toxin, stops bleeding and prevents miscarriage.	3–10
*Gan Cao (Radix glycyrrhizae)*	15 (26.3)	Acute hepatic necrosis (3), Acute hepatitis (8), Mixed hepatocellular-cholestatic hepatitis (1), Enzyme elevations without jaundice (3)	8.9 (10.6)	Strengthens spleen and improves 'qi', clears heat and removes toxin, dispels phlegm in order to relieve cough, relax spasm and relieves pain and moderate drug actions.	2–10
Ze Xie (*Alisma orientalis*)	14 (24.6)	Acute hepatic necrosis (2), Acute hepatitis (11), Mixed hepatocellular-cholestatic hepatitis (1)	5.9 (14.4)	Promotes urination to drain dampness, discharge heat, revolves turbidity, and lowers lipid	6–10
Chuan Xiong (*Rhizoma ligustici*)	14 (24.6)	Acute hepatic necrosis (1), Acute hepatitis (10), Mixed hepatocellular-cholestatic hepatitis (1), Enzyme elevations without jaundice (2)	6.3 (8.3)	Activates blood and moves 'qi', and dispels wind in order to relief pain.	3–10

Total number of reports included in data analysis, *N* = 57

Abbreviations used: *TCM* Traditional Chinese Medicine, *TDD* total daily doses of raw herb (in grams)

^a^ Information is obtained from the Pharmacopoeia of the People’s Republic of China, 9th Ed, 2010 (English Ed)

The distribution of the type of hepatotoxicity reported is shown in Table 4. Majority (59.6 %) of the cases reported acute hepatitis as the main adverse event. Cholestatic hepatitis, on the other hand, was seen only in three (5.3 %) cases. There were no reports of hepatic adenoma or hepatocellular carcinoma. In general, CAMs were used by these patients to promote health and well-being (6 cases), for slimming and weight control (9 cases), and minor ailments (22 cases) such as cough, gastrointestinal complaints and pain relief. Five patients used CAM for liver-related conditions e.g. jaundice, abdominal distension; and strengthening of liver function. Among herbal ingredients which were previously reported in the literatures as possible hepatotoxic agents [37–53], some of those found were Chai Hu (*Radix bupleuri*) (11 cases), Da Huang (*Radix et rhizoma rhei*) (6 cases), Chuan Lian Zi (*Fructus toosendan*) (2 cases), Hu Zhang (*Radix polygoni cuspidati*) (2 cases), and He Shou Wu (*Radix polygoni multiflori*) (1 case), and are elaborated in Table 5.

**Table 4:** Phenotypes of hepatotoxicity reported with the corresponding CAM implicated and outcome of AE

Phenotypes of DILI ^a^	No. of cases, n (%)	Type of CAM implicated (n)	Outcome of AE (n)
Acute hepatitis	34 (59.6)	TCM (22), Health supplements (7), Other traditional medicines (5)	Death (2), Not recovered (21), Recovered (7), Uncertain outcome (4)
Enzyme elevations without jaundice	9 (15.8)	TCM (6), Health supplements (3)	Not recovered (5), Recovered (2), Uncertain outcome (2)
Acute hepatic necrosis	5 (8.8)	TCM (4), Health supplements (1)	Death (1), Recovered (1) Not recovered (3)
Cholestatic hepatitis	3 (5.3)	TCM (2), Health supplements (1)	Death (1), Not recovered (1), Recovered (1)
Mixed hepatocellular-cholestatic hepatitis	3 (5.3)	TCM (1), Health supplements (1), Other traditional medicines (1)	Not recovered (2), Recovered (1)
Chronic hepatitis	2 (3.5)	Health supplements (2)	Death (1), Not recovered (1)
Nonalcoholic fatty liver	1 (1.8)	Health supplements (1)	Uncertain outcome (1)

Total number of reports included in data analysis, *N* = 57

*AE* adverse event, *CAM* complementary and alternative medicine, *DILI* drug-induced liver injury, *TCM* Traditional Chinese Medicine

^a^ Phenotypes of hepatotoxicity were adapted from the NIDDK LiverTox Database [34]

**Table 5:** Suspected herbal ingredients with previous documented reports of possible hepatotoxicity

Name of herbal ingredient	No. of cases, n (%)	Types of hepatotoxicity implicated (n)	Mean TDD (g) (SD)	Mechanisms of action (based on TCM system of therapeutics) ^a^	Recommended daily doses (g) ^a^
Chai Hu (*Radix bupleuri)*	11 (19.3)	Acute hepatitis (9), Acute hepatic necrosis (2)	18.0 (33.5)	Disperses and reduces fever, soothes the liver in order to alleviate mood, increase ‘yang qi’	3–10
Cang Zhu (*Atractylodes lancea*)	8 (14.0)	Acute hepatitis (3), Acute hepatic necrosis (2), Cholestatic hepatitis (1), Enzyme elevations without jaundice (1), Mixed hepatocellular-cholestatic hepatitis (1)	7.9 (15.5)	Reduces ‘dampness’, strengthens the spleen, dispels ‘wind’ and dissipates ‘cold’, and improves vision	3–9
Zhi Ban Xia (*Rhizoma pinelliae ternatae preparata)*	7 (12.3)	Acute hepatitis (3), Acute hepatic necrosis (2), Enzyme elevations without jaundice (2)	5.7 (3.4)	Reduces ‘dampness’ and phlegm, suppresses counteractive flow (e.g. vomiting), dissolves lumps and reduces masses	3–9
Xi Xin (*Radix et rhizome asari*)	7 (12.3)	Acute hepatitis (4), Acute hepatic necrosis (1), Enzyme elevations without jaundice (2)	2.2 (1.6)	Dispels ‘wind’ and dissipates ‘coldness’, dispels ‘wind’ and relieves pain, relieves blocked nose, warms the lungs and resolves fluid retention	1–3
Da Huang (*Radix et rhizoma rhei*)	6 (10.5)	Acute hepatitis (3), Acute hepatic necrosis (1), Enzyme elevations without jaundice (1), Mixed hepatocellular-cholestatic hepatitis (1)	8.1 (15.2)	Removes accumulation of waste materials through purging, clears ‘heat’ and purges ‘fire’, cools the blood and removes toxins, expel stasis in order to unblock the meridians, drains ‘dampness’ to reduce jaundice	3–15
Ma Huang (*Ephedra sinica*)	4 (7.0)	Acute hepatitis (2), Acute hepatic necrosis (1), Enzyme elevations without jaundice (1)	3.7 (1.2)	Promotes sweating and dissipates ‘cold’, diffuse the lungs to relieve panting, and promotes urination to alleviate edema	2–10
Tian Hua Fen (*Radix trichosanthis*)	3 (5.3)	Acute hepatitis (2), Cholestatic hepatitis (1)	1.1 (1.3)	Clears ‘heat’ and purges ‘fire’, gives rise to fluid to quench thirst, disperses swelling and expels pus	10–15
Bai Xian Pi (*Dictamni cortex*)	2 (3.5)	Acute hepatic necrosis (1), Acute hepatitis (1)	0.7 (N/A ^b^)	Clears ‘heat’ and dry ‘dampness’, dispels ‘wind’ and removes toxins	5–10
Jue Ming Zi (*Semen cassiae*)	2 (3.5)	Acute hepatitis (1), Enzyme elevations without jaundice (1)	0.7 (0.7)	Clears ‘heat’ and improves vision, ‘moistens the intestines to promote bowel movement	9–15
Chuan Lian Zi (*Fructus toosendan*)	2 (3.5)	Acute hepatitis (2)	1.2 (N/A ^b^)	Soothes the liver and discharges ‘heat’, move ‘qi’ to relieve pain, kill worms	5–10
Cang Er Zi (*Fructus xanthii sibirici*)	2 (3.5)	Acute hepatitis (2)	4.7 (4.7)	Disperses coldness caused by wind, relieves blocked nose, dispels ‘wind-dampness’	3–10
Hu Zhang (*Radix polygoni cuspidati*)	2 (3.5)	Acute hepatitis (2)	4.6 (4.8)	Reduces ‘dampness’ to abate jaundice, clears heat and removes toxins, dissipates stasis and relieves pain, suppresses cough and resolves phlegm	9–15
Du Huo (*Radix angelicae pubescentis*)	2 (3.5)	Acute hepatic necrosis (1), Acute hepatitis (1)	1.9 (1.6)	Dispels ‘wind’ and removes ‘dampness’, relieves pain	3–10
He Shou Wu (*Radix polygoni multiflori*)	1 (1.8)	Acute hepatitis (1)	1.0 (N/A)	Removes toxins, disperses abscesses, manage malaria, and ‘moistens’ the intestines to promote bowel movement	3–6
Zhi He Shou Wu (*Radix polygoni multiflori praeparata)*	1 (1.8)	Acute hepatitis (1)	1.8 (N/A)	Strengthens the liver and kidney, replenishes essence and blood, darkens beard and hair, strengthens sinew and bone, and reduces lipid levels	6–12

Total number of reports included in data analysis, *N* = 57

Abbreviations used: *TCM* Traditional Chinese Medicine, *TDD* total daily doses of raw herb (in grams)

^a^ Information is obtained from the Pharmacopoeia of the People’s Republic of China, 9th Ed, 2010 (English Ed)

^b^ Standard deviations for Bai Xian Pi (*Dictamni cortex*) and Chuan Lian Zi (*Fructus toosendan*) were not available due to insufficient dosing information

Twenty-nine (82.9 %) of the 35 cases had sufficient information for the computation of a RUCAM score. The median score for these reports was 4, with a range of 1 to 8. The interpretation of the scores are as follows: 0 or less indicates that the drug is “excluded” as a cause; 1 to 2 that it is “unlikely”; 3 to 5 “possible”; 6 to 8 “probable”; and greater than 8, “highly probable”. The RUCAM score for the remaining 6 cases were not available due to a lack of essential information, such as the time to onset, transaminase levels and whether any exclusion tests e.g. viral markers, antibody titres, were performed to rule out alternative causes of hepatotoxicity.

Concurrent use of western medicines was also reported in 30 (52.6 %) of the cases, where 16 (53.3 %) of them contained active pharmaceuticals that are potentially hepatotoxic. Some of these examples are the statins, fibrates, paracetamol, and phenytoin. The RUCAM scores were calculated for 10 (62.5 %) of these 16 cases, and the median was 1 (range: 0 to 5).

## Discussion

Through this study, the patterns of hepatotoxicity associated with the use of CAM were analysed over a 6-year period from 2009 to 2014. In general, an average of 140 cases related to CAM was reported yearly, and about 9 % of these involved hepatotoxicity.

### Demographic information of the adverse event reports

There was no difference in the gender distribution of the AERs. This is opposed to previous findings that women were more likely to be affected by adverse reactions to drug treatment [39, 54, 55]. Differences in endogenous and exogenous hormones, body size and fat compositions, and also liver metabolism were cited as possible reasons for the gender differences. A woman’s reproductive function may also increase the need for medications at an earlier age than men, increasing the likelihood of adverse drug reactions [55]. In this study, the small sample size of 57 reports may be insufficient to conclude whether there are any gender differences predisposing to hepatotoxicity. Furthermore, only one patient used CAM to regulate menstruation, and none for contraceptive or reproductive purposes.

Majority (73.7 %) of the patients were above 40 years of age, and there was no significant differences (*p* = 0.755) in the gender distribution in this age group (19 males, 23 females). Age has been cited as a risk factor for hepatotoxicity due to the increased likelihood of comorbid conditions and deteriorating organ function, especially the liver [41, 56]. Therefore it is not unexpected that a large proportion of cases involved patients above 40 years old. Nonetheless, under-reporting of DILI is still a prevalent phenomenon in many countries [57, 58] and the demographic characteristics of patients in this study may not accurately reflect those found in prospective cohort studies of DILI.

### Types of CAM implicated and outcomes of adverse events

The proportions of different types of CAM used were similar to other studies conducted in Singapore [17, 59, 60]. TCM accounted for the majority of CAM usage, followed by health supplements, regardless of ethnicity.

TCM, was implicated in 61.4 % of the adverse events. The major affected ethnic group was the Chinese (82.5 %). These findings are congruent with the earlier review of AERs associated with complementary medicines and health supplements [21]. The study found that CPMs and Chinese patients were more commonly implicated in adverse events than other CAMs and ethnic groups. The reason for these similar findings could be attributed to the widespread availability and relatively easy accessibility of TCM products in Singapore where the population is 80 % of Chinese ethnicity.

### Assessment of DILI cases involving TCM

Among the 35 cases involving TCM products, RUCAM scores were calculated for 29 (82.9 %), with a median score of 4 and range of 1 to 8. Only Chinese herbal ingredients that were previously reported in the literature as possibly risky for liver injury (Table 5) were assessed for causality. The range of scores indicates causalities of unlikely, possible or probable. None of these cases had a score of 0 or less, which suggests that the likelihood of hepatotoxicity for these TCM cannot be entirely ruled out. Notwithstanding the range of scores obtained for these 29 cases, a proper evaluation of causality for these DILI cases was challenging and hard to ascertain.

In addition, most of the patients used at least ten different ingredients, and the duration of use ranged from a few months to 3 years. The use of multiple products over an extended period of time could have produced a heavy metabolic burden on the liver. This could predispose the patients to acute liver injuries, regardless of the presence of potentially hepatotoxic ingredients.

Chinese herbal ingredients taken at recommended doses by the Chinese Pharmacopoeia typically do not present harm to the liver, though it is unclear whether prolonged use or abrupt changes in the dosage can precipitate hepatotoxicity. In this study, most of the TCM ingredients were used within their recommended dose ranges. There were two patients that used a number of ingredients beyond their recommended daily doses; some examples were Dang Gui (*Radix angelicae sinensis*), Dang Shen (*Radix codonopsis*) and Chai Hu (*Radix bupleuri*). Among these ingredients, only Chai Hu was previously reported in the literature as potentially hepatotoxic [37, 43, 47, 61]. However, due to the advanced age of both patients (70 and 83 years old), coupled with the use of numerous herbal products over a prolonged period, it was hard to pinpoint specific agents that could have caused DILI.

Accurate naming of the correct plant species and specific plant part is also crucial for an appropriate assessment. Unfortunately, AERs rarely provide sufficient details on the plant family, subfamily, species, subspecies or variety used. This specification is necessary as hepatotoxic chemicals may be distributed unevenly throughout some plants [62]. For example, if rhizomes of a plant are prescribed, additional clarification is needed on whether the peeled or unpeeled rhizome was used, the extent of peeling, as well as whether any adjacent parts were included in the herbal product. The degree of processing may also influence the toxicity of a plant species, such as He Shou Wu (*Radi polygoni* multiflori), where the processed form (Zhi He Shou Wu [*Radix polygoni multiflori praeparata*]) would be less hepatotoxic and higher daily doses could be recommended. Variations in these aspects could have affected the toxicity potential of different formulations, and in turn influence the outcome of causality assessments [44].

With the regulation of CPMs in Singapore, many of these factors influencing the accuracy of herbal hepatotoxicity assessments can be mitigated to a certain extent. The processes of listing CPM products for regulatory approval ensures manufacturers and importers accurately report the specific ingredients and amounts within each product [63]. Requirements on labelling, prohibited claims, types of test reports, quality parameters are needed before a CPM can be listed.

As no CPM is 100 % safe, DILI can occur with no plausible explanations. For example, the Chinese herbal plant Chai Hu (*Radix bupleuri*) is traditionally used to treat liver-related conditions such as jaundice, and is said to disperse liver ‘qi’ stagnation and reduce dampness. However, Chai Hu had been implicated in multiple cases of acute hepatitis both as an ingredient alone and within a particular formulation “Xiao Chai Hu Tang” (also known as Syo-Saiko-To in Japanese) [37, 43, 47, 61]. The incidences had been linked to the consumption of Chai Hu for prolonged periods of time, even at therapeutic doses, and evaluated as possible to probable causality. Additionally, the mentioned reports ruled out other possible causes of liver injury, as well as any quality and manufacture issues of the Chai Hu-containing products. In this study, 11 cases with Chai Hu as one of the suspected ingredients were identified. The mean TDD was 18.0 ± 33.5 g, which was more than the Chinese Pharmacopoeia recommended range of 3 to 10 g. However, there was a large variability in the TDD among the 11 cases, with minimum and maximum daily doses of 0.2 g and 100 g respectively. Differences in the clinical course and presence of confounders in the cases could have complicated the evaluation process and made it difficult to establish a general observation. Therefore, the likelihood of hepatotoxicity with Chai Hu could not be adequately addressed.

Another patient, where a causality assessment of “certain” was assigned, experienced abdominal pain, poor appetite, dark coloured urine and malaise for about 10 days, after taking TCM for about 1 month. She was advised to discontinue the TCM products because of the possibility of DILI. About 6 months later, she took TCM once again and developed liver injury after 3 days of consumption. Investigations did not establish any abnormal aetiology and the hepatitis resolved after supportive treatment. The temporal relationship on multiple occasions, along with the exclusion of other possible causes, gave a strong indication of possible TCM-induced DILI. However, due to the wide range of herbal ingredients which the patient was taking, it was difficult to pinpoint any exact causative agents, making any conclusive assessment highly challenging.

DILI remains poorly understood and are often neglected. The limited body of knowledge presents a major clinical and regulatory challenge for appropriate assessment of causal factors. Although expert opinion from clinicians and regulatory authorities remain the mainstay for conducting evaluations, more objective methods such as probability scales and mathematical models may be necessary to supplement causality evaluation [24]. A recent review of systematic reviews involving DILI associated with CAM had shown the importance of including case reports and series in these reviews, in order to obtain a more holistic interpretation of the available evidence [64].

### RUCAM Scores for concomitant western medicines

It was necessary to rule out the influence of western pharmaceuticals with reported hepatotoxicity in the literature. Sixteen out of the 57 cases were reviewed for confounding effects by western medicines. The RUCAM was used for 10 of these 16 cases, and the median score was 1 (range: 0 to 5). RUCAM scores were not computable for the remaining 6 cases due to a lack of information. Among the 10 cases, there was only one case with a score of 5, while the scores for the other 9 were from 0 to 2. This means that for most of the cases, causality for the western medicines were either excluded or unlikely. From these observations, it was unlikely that the western medicines used attributed to the adverse reactions reported.

### Limitations of the study

#### Lack of necessary information

The retrospective design of this study limited the amount of information that could be collected and analysed. Details of the indication of CAM products, posology and administration, actual periods of use and other necessary information were often incomplete or even lacking. Furthermore, most of the patients were on multiple types of CAM. Reporters or patients might also have been unwilling to share certain information they deemed sensitive. Poor monitoring of administration timelines by patients, in particular the elderly were likely (28.1 % of the cases were above 60 years old). In addition, product information such as the source and manufacturer details was missing in some cases. For TCM products that are not regulated as CPMs, specifications on the plant parts used and the processing or preparation methods were lacking. The poor standardisation of information across the 57 reports made it challenging to analyse them under a common denominator. This could lead to a misinterpretation or oversimplification of the data obtained.

#### Presence of confounders

The issue of insufficient information was further compounded by the presence of multiple confounders in the patients, such as pre-existing liver diseases, alcoholism, and the use of western medicines, which could have influenced the causality assessment of hepatotoxicity to a certain extent. Under-reporting is also commonly present in spontaneous reporting systems, especially with the use of CAM [40, 58]. Many patients might not have regarded CAM as a “medicine” or might have been reluctant to share information on such use with their health care providers. Furthermore, most CAM products were available over-the-counter and patients need not seek the advice of health professionals before using them.

#### Drug-herb interactions

The methodology of this study might have not been robust enough to pick up potential drug-herb, herb-herb, or herb-food interactions, especially if patients were reluctant to share or neglected the importance of such information. The presence of interactions could confound the assessment of hepatotoxicity in patients, as many drugs/herbs were orally ingested and hence subjected to hepatic first-pass. This could potentially lead to an over- or underestimation of the causality of certain drugs or herbs. It was therefore crucial to obtain a complete picture of a patient’s pharmacotherapy in order to appropriately assess DILI. Prospective designs could be explored to evaluate individual herbs and their respective interactions.

#### Review of non-TCM CAM

As majority (61.4 %) of the adverse events involved TCM, the primary analyses mainly looked at the different Chinese herbal ingredients and their hepatotoxic potential. Information regarding the other forms of CAM which include but are not limited to the Malay Jamu, Indian Ayurveda and the Perso-Arabic Unani medicine were limited and hence the study was unable to thoroughly evaluate the risk of hepatotoxicity for these fields of medicine. Although Malay Jamu and Indian Ayurveda constituted 10.5 % of the cases in the study, most of the ingredients either could not be identified accurately or were largely dissimilar and no particular ingredients stood out amongst the cases. Nonetheless, further reviews could be conducted specifically for these groups of CAM to detect potential signals of hepatotoxicity or other adverse reactions.

## Conclusions

In this study, we outline the patterns of hepatotoxicity associated with the use of CAM through a review of AERs. In addition, AERs associated with TCM products were looked at in detail, and Chinese herbal ingredients with a high propensity for hepatotoxicity were highlighted. Patients who used CAM generally did not anticipate any risks involved with their therapy, and were not strictly compliant at all times. Majority of the health professionals were unable to evaluate the cases adequately due to insufficient information provided by the patients, as well as the lack of extensive clinical evidence base for the CAM therapies.

The findings suggest the need for improvement on prospective evaluations of suspected DILI cases, preferably with the aid of structured causality assessment tools. In the modern era of patient-centred care, the notion of “evidence-based medicine” is highly crucial. Clinical trials have been increasingly conducted for CAM over the past decades [65–69], and the efficacy of herbs such as ginger (*Zingiber officinale*) and various ginseng species has been well-established [66, 69]. Healthcare professionals play an important role in spontaneous ADR reporting and monitoring the safety of CAM used by the population [70]. Their interactions with the AE reporting system to facilitate collection, monitoring and evaluation of adverse drug events are highly crucial. Lastly, similar analyses could also be conducted to evaluate the risk-benefit profiles of CAM involving other organ systems.

## Abbreviations

ADR, adverse drug reaction; AE, adverse event; AERs, adverse event reports; ART, adverse reaction terminology; CAM, Complementary and Alternative Medicine; CHP, complementary health products; CFDA, China Food and Drug Administration; CMIS, Critical Medical Information Store; CPM, Chinese Proprietary Medicines; DILI, drug-induced liver injury; EMRX, Electronic Medical Record Exchange; HSA, Health Sciences Authority; NIDDK, National Institute of Diabetes and Digestive and Kidney Diseases; NUS-IRB, National University of Singapore Institutional Review Board; PRISM, Pharmaceutical Regulatory Information System; RUCAM, Roussel Uclaf Causality Assessment Method; TDD, total daily dose; TCM, Traditional Chinese Medicine; UMC, Uppsala Monitoring Centre; VCB, Vigilance and Compliance Branch; WHO, World Health Organisation
